# Comprehensive analysis of anoikis-related gene signature in ulcerative colitis using machine learning algorithms

**DOI:** 10.3389/fmed.2025.1498864

**Published:** 2025-03-06

**Authors:** Peng Liu, Chunyan Sun, Xiaojuan Wang, Bing Han, Yuhao Sun, Yanbing Liu, Xin Zeng

**Affiliations:** ^1^Department of Gastroenterology, Shanghai East Hospital, Tongji University School of Medicine, Shanghai, China; ^2^Department of Pharmacy, Minhang Hospital, Fudan University, Shanghai, China

**Keywords:** ulcerative colitis, anoikis, diagnostic marker, machine learning algorithm, immune cells

## Abstract

Ulcerative colitis (UC) is a chronic inflammatory bowel disease with an idiopathic origin, characterized by persistent mucosal inflammation. Anoikis is a programmed cell death mechanism activated during carcinogenesis to eliminate undetected isolated cells from the extracellular matrix. Although existing evidence indicates that anoikis contributes to the modulation of immune response, the involvement of anoikis-related genes (ARGs) in UC pathogenesis and their interaction with infiltrating immune cells has not been thoroughly explored. The GSE75214, GSE92415, and GSE16879 datasets were acquired and integrated from the GEO database. Additionally, 58 ARGs were identified through the GSEA database. Key anoikis-DEGs in UC were identified using three machine learning algorithms, including least absolute shrinkage and selection operator (LASSO) Cox regression, random forest (RF), and support vector machine (SVM). Receiver operating characteristic (ROC) analysis was utilized to evaluate the diagnostic accuracy of each gene. Subsequently, Single sample GSEA (ssGSEA) was executed to explore the relationships within immune cell infiltration, UC subtypes, and key anoikis-DEGs. Besides, unsupervised cluster analysis was conducted to categorize the UC samples into distinct subgroups, followed by comparing subtype differences. Finally, the upstream regulatory network was constructed and visualized. A comprehensive analysis of the involvement of ARGs in UC was performed, revealing their expression profile, correlation with infiltrating immune cells, and enrichment analyses. We identified five key anoikis-DEGs (*PDK4*, *CEACAM6*, *CFB*, *CX3CL1*, and *HLA-DMA*) and demonstrated their high diagnostic accuracy for UC. Moreover, *CEACAM6*, *CFB*, *CX3CL1*, and *HLA-DMA* exhibited positive associations with infiltrating immune cells in UC, whereas *PDK4* displayed a negative correlation with all immune cells. Unsupervised cluster analysis enabled the classification of UC patients into two clusters, both of which exhibited distinct gene expression profiles and immune signaling pathways. Further, based upon the upstream regulatory network, TP53, RARB, RXRB, and CTCF potentially exerted regulatory functions. Our analysis identified five key anoikis-DEGs as characteristic biomarkers of UC. These genes were strongly associated with the infiltration of both innate and adaptive immune cells, as well as immune pathways. This study highlights the role of anoikis genes in UC pathophysiology and offers valuable insights for further elucidating UC pathogenesis and individualized therapy.

## 1 Introduction

Ulcerative colitis (UC), a primary subtype of inflammatory bowel disease (IBD), exhibits continuous colonic mucosal inflammation that extends proximally from the rectum (1, 2). This disease affects nearly 5 million individuals worldwide in 2023, with its global disease burden on the rise (3). In China, the reported incidence of UC is 3.35 per 100,000 males and 2.65 per 100,000 females (4). Early diagnosis is critical for effective UC treatment, yet specific diagnostic signatures are lacking. At present, the biomarkers for UC include serum anti-αvβ6 antibodies and serum oncostatin M in diagnosing, fecal calprotectin and serum trefoil for disease activity assessment, as well as whole blood transcriptomic panels and CLEC5A/CDH2 ratio for determining the need for escalated treatment (5). In addition, despite recent advancements in diagnostic and therapeutic methods, including surgical and immunotherapeutic strategies, the prognosis for UC remains unsatisfactory. A growing body of evidence has identified that UC is frequently driven by abnormalities in a wide range of immune cell types, encompassing leukocytes, macrophages, B cells, dendritic cells, and both regulatory and effector T cells (6, 7). However, the multifaceted pathophysiology and immune mechanisms underlying UC are not completely understood. Consequently, the identification of novel characteristic genes has the potential to accelerate the unveiling of prospective therapeutic targets, innovative intervention approaches, and a deeper understanding of the pathogenic mechanisms and etiology of UC. This, in turn, can enable the implementation of more successful strategies for diagnosis and therapy.

Anoikis, a form of programmed cell death, occurs upon the detachment of cells from their designated extracellular matrix (ECM). Its primary role lies in preventing dysplastic cell growth or improper matrix attachment (8, 9). Anoikis regulates anchorage-dependent growth and epithelial-mesenchymal transition, thus being indispensable for preserving tissue homeostasis and proper development. Additionally, it serves as a significant regulatory factor in the context of metastatic cancers, cardiovascular diseases, and diabetes. Molecular pathways and mechanisms that affect anoikis resistance have been revealed in recent years (8, 10). These pathways include a range of factors, such as growth factors, cell adhesion molecules (CAMs), and pathways with the capacity to induce epithelial-mesenchymal transitions. The complex network of downstream molecules includes focal adhesion kinase (11), Src kinase (12), mitogen-activated protein kinase (MAPK) (13), ERK1/2 (14), Bcl-2 family (15), PI3K/Akt (16, 17), and insulin-like growth factor receptors (18). Each of these pathways is crucial for preventing cell death and promoting cell survival. In recent years, studies have shown various factors influencing anoikis, comprising integrins, IGFR, EGFR, TGF-*β*, Trk, NF-κB, E-calmodulin, the Hippo pathway, eEF-2 kinase, ROS, acidosis, hypoxia, and protective autophagy (8, 10). Cell death has the potential to directly or indirectly compromise barrier function and impede epithelial restitution in IBD. The primary pathological damage of UC results from dysregulated immune responses induced by commensal microflora. This dysregulation leads to the production of inflammatory cytokines, the infiltration of lymphocytes from the bloodstream into the inflamed bowel, and innate immune cell signaling (19).

Currently, therapeutic strategies for UC include anti-adhesion therapy (20), anti-TNF monoclonal antibodies (21, 22), anti-IL-12/IL-23p40 antibodies (23), and JAK inhibitors (24). Therefore, anoikis may play a crucial role in the onset and progression of UC. Multiple scientific studies have robustly confirmed the integral role of anoikis in the pathogenesis of diverse diseases, with a particular emphasis on its significance in tumor immunity. These studies have explored a wide range of conditions, including glioblastoma (25), head and neck squamous cell carcinoma (26), and lung adenocarcinoma (27). A previous study demonstrated that nuclear MYH9 bound to the CTNNB1 promoter and promote CTNNB1 transcription, thereby conferring resistance to anoikis in gastric cancer (28). Moreover, the formation of a protein complex comprising Bim-EL, LC8, and Beclin-1 might contribute to the evasion of anoikis in inflammatory breast cancer (29). In another study by Jin et al., it has been verified that GDH1-mediated metabolic reprogramming of glutaminolysis promotes resistance to anoikis and tumor metastasis in LKB1-deficient lung cancer (30). In addition, a study revealed that *Aloe vera* gel polysaccharide (AGP) induced Nrf2 activation, reduced ROS levels, ameliorated mitochondrial dysfunction, and alleviated anoikis caused by impaired-mitochondrial function of colonic epithelial cells to maintain intestinal barrier integrity in DSS-induced colitis mice (31). Despite increasing evidence indicating that anoikis contributed to UC progression, its predictive value and association with immune response regulation in UC remains unexplored. Similarly, the underlying mechanisms associated with anoikis in the manifestation and progression of UC remain unknown. Thus, a comprehensive study is warranted to investigate differences in immune characteristics between normal tissues, UC specimens, and different UC subtypes. This research is vital for elucidating the cellular features and molecular mechanisms underlying anoikis and its associated genes. Additionally, establishing characteristics related to anoikis can offer valuable insights for the individualized treatment of UC patients.

A thorough and multiscale bioinformatics analysis was executed to determine the differentially expressed genes (DEGs) in samples from UC patients and normal individuals, focusing on anoikis-related genes (ARGs) and immune infiltration profiles. Moreover, the enrichment analyses were performed to investigate the differences between UC and normal samples. Subsequently, anoikis-DEGs were identified to explore the association between anoikis and UC. Using three machine learning algorithms, we finally obtained five risk signature genes for their potential to predict disease onset. Immune infiltration and functional enrichment analyses were also performed using these five key anoikis-DEGs. Simultaneously, an unsupervised clustering analysis and functional enrichment analysis was executed to distinguish differences between various clusters. Further, gene-miRNA and gene-transcription factor regulatory network associated with the five risk genes were assessed. In summary, our findings offer a comprehensive insight into the connection between anoikis and UC, establishing a foundation for individualized diagnosis and management of UC.

## 2 Materials and methods

### 2.1 Data sources and processing

The gene expression profiling data for UC, encompassing GSE75214, GSE92415, and GSE16879 datasets, were retrieved from the gene expression omnibus (GEO) database.^1^ The GSE75214 dataset included gene expression profiles from 97 individuals with UC and 11 controls, while the GSE92415 dataset comprised gene expression profiles from 87 pre-treatment UC patients and 21 healthy individuals. The GSE16879 dataset featured gene expression data from 24 pre-treatment UC patients and six control healthy volunteers.

Samples unrelated to this study were excluded, resulting in 246 samples included: 208 UC mucosal biopsy samples and 38 normal samples. The merged dataset was subjected to batch correction utilizing the “limma” and “sva” packages (32). Principal component analysis (PCA) was employed to evaluate the effectiveness of batch effect removal and to visualize the distribution of samples from UC and healthy individuals. Furthermore, the merged data underwent normalization utilizing the R package “preprocessCore.” Subsequently, 58 ARGs were extracted from the molecular signatures database,^2^ as illustrated in Supplementary Table 1.

### 2.2 Identification of DEGs and functional enrichment analysis

Identification of DEGs was carried out by the “limma” R package, ensuring the criteria of |log_2_ fold change (FC)| > 0.5 and adjusted *p*-value <0.05 (33). Volcano plots were created using the R packages “limma” and “ggplot2” to show the distribution of DEGs. The “ComplexHeatmap” R package was employed to visualize the top 20 upregulated and downregulated genes. The Gene ontology (GO) and Kyoto Encyclopedia of Genes and Genomes (KEGG) enrichment analyses were determined on the merged DEGs utilizing the R package “clusterProfiler” to explore differential biological pathways related to the signature genes. Statistically significance was defined as *p* < 0.05.

### 2.3 Identification of anoikis-DEGs in UC and functional enrichment analysis

The overlapping genes between the upregulated and downregulated DEGs of UC and ARGs were assessed *via* the “limma” R package. Additionally, the GO and KEGG pathway enrichment analysis was conducted to further analyze the signaling pathways of anoikis-DEGs. Statistically significance was defined as *p* < 0.05.

### 2.4 Differential gene expression analysis of ARGs between UC and healthy individuals

To present a thorough overview of the differential expression of ARGs in UC and healthy individuals, the differential expression levels of ARGs were visualized in volcano plots and heatmap using the R packages “limma,” “ggplot2,” and “ComplexHeatmap.” Subsequently, we explored the expression of 21 anoikis-DEGs in UC and normal samples. Simultaneously, a protein–protein interaction (PPI) network analysis was conducted on 21 anoikis-DEGs utilizing the STRING database (https://string-db.org/) and displayed using the Cytoscape 3.9.0 software.

### 2.5 Identification of key Anoikis-DEGs in UC using machine learning

Three machine learning algorithms were utilized in this study to identify the key anoikis-DEGs as characteristic biomarkers in UC. The first algorithm employed was the least absolute shrinkage and selection operator (LASSO) logistic regression by using the R package “glmnet” (34). LASSO was selected for its ability to perform both variable selection and regularization, which helps identify important genes while reducing overfitting, especially in high-dimensional datasets. To ensure robustness and avoid overfitting of the model, we employed 10-fold cross-validations to determine the parameter *λ*. According to the optimal λ value and the corresponding coefficients, nine genes were obtained. The second algorithm was the random forest (RF) algorithm, assessed utilizing the R package “randomForest” to grow a forest consisting of 500 trees with default settings (35). RF algorithm, an ensemble learning method, was chosen for its robustness and ability to construct multiple decision trees and aggregate their predictions to improve accuracy and prevent overfitting. Using the RF algorithm, the top 10 genes with the highest importance were selected for downstream analysis. Finally, the support vector machine (SVM) algorithm was utilized, with the analysis conducted using the R package “kernlab” (36). The SVM algorithm can rank features based on recursion to avoid overfitting, and 13 genes were obtained through this approach. The five characteristic biomarkers for diagnosis were finally identified as the common core genes among the three algorithms. Furthermore, the five key anoikis-DEGs’ interactions were illustrated using the “chordDiagram” R package. Subsequently, the ROC curves and the area under the curve (AUC) were obtained utilizing the R package “pROC.”

### 2.6 Validation of the key anoikis-DEGs in colitis animal model

Animal experiments were conducted in accordance with the ethical requirements and approved by the Tongji University Animal Ethics Committee. The C57BL/6 male mice were randomly allocated into normal control and colitis groups. The Control healthy mice were provided normal water. The colitis model was induced by administering 3.5% dextran sulfate sodium (DSS, Yeasen Biotechnology, 60316ES76, China) for 7 days, followed by 3 days of regular drinking water. Then, the mice colon tissues were obtained for RNA extraction to quantify the gene expression of the key anoikis-DEGs using Quantitative Real-time PCR (qPCR). Tissue total RNA was extracted using Trizol reagent (Invitrogen, 15,596,018, United States) and reverse to cDNA using an Evo M-MLV kit (Accurate Biology, AG11705, China). The Primer sequences were obtained from PrimerBank^3^ and synthesized at Sangon Biotech (Shanghai, China). qPCR was performed using the SYBR Green Pro Taq HS premix qPCR (Accurate Biology, AG11701, China) on a SLAN-96S Real-Time PCR System. The results were replicated three times, and the levels of target genes were normalized to GAPDH and analyzed utilizing the 2^−(⊿⊿Ct)^ method. *CEACAM6* is not present in rodents, and *H2-DMa* is orthologous to human *HLA-DMA*. Thus, we analyzed the expression of four key anoikis genes: *PDK4*, *H2-DMa*, *CX3CL1*, and *CFB*. The following is a list of the primer sequences (5′-3′):

PDK4-F AGGGAGGTCGAGCTGTTCTC.

PDK4-R GGAGTGTTCACTAAGCGGTCA.

H2-DMa-F CTCGAAGCATCTACACCAGTG.

H2-DMa-R TCCGAGAGCCCTATGTTGGG.

CX3CL1-F ACGAAATGCGAAATCATGTGC.

CX3CL1-R CTGTGTCGTCTCCAGGACAA.

CFB-F GAGCGCAACTCCAGTGCTT.

CFB-R GAGGGACATAGGTACTCCAGG.

GAPDH-F TGGCCTTCCGTGTTCCTAC.

GAPDH-R GAGTTGCTGTTGAAGTCGCA.

### 2.7 The immune infiltration characteristics in UC samples and the association between key anoikis-DEGs and infiltrating immune cells

Single sample GSEA (ssGSEA) is an extension of the GSEA method that enables the assessment of infiltrated immune cells and the activity of specific immune factors (37). ssGSEA was performed to measure the correlation coefficients between immune infiltration cells using the “GSVA” R package. Pearson’s correlation coefficient analysis was utilized to investigate the association between immune infiltrating cells. Heatmaps were generated using the R package “ggplot2.” Simultaneously, the “ggplot2” package was utilized to display immune cell infiltration as box plots in UC and healthy volunteers, with Pearson’s correlation coefficient evaluating relationships between different infiltrating immune cells. The association between the key anoikis-DEGs and immune infiltrating cells was assessed utilizing Pearson’s analysis and visualized in Lollipop plots using the R package “ggplot2.”

### 2.8 Correlation of key anoikis-DEGs with all UC genes and GSEA analysis

Next, the correlation of the key anoikis-DEGs with all UC genes was assessed, and a heatmap was utilized to display the 50 leading positively correlated genes. Additionally, Reactome pathway analysis was performed using GSEA for each diagnostic marker utilizing the R package “clusterProfiler” to explore the top 20 associated biological pathways.

### 2.9 Identification of anoikis-related subtypes in UC and functional enrichment analysis

To explore the functional role of ARGs in UC, unsupervised cluster analysis was performed utilizing the R package “ConsensusClusterPlus” as per the key anoikis-DEGs (38). Moreover, the differential expression of key anoikis-DEGs among different subtypes were explored. Moreover, a heatmap was generated utilizing the R package “pheatmap” to demonstrate the association between clinical features, gene expression, and subtypes.

Three gene sets (“h.all.v7.5.1.symbols.gmt,” “c2.cp.kegg.v7.5.1symbols,” and “c2.cp.reactome.v7.5.1.symbols”) were acquired from the MSigDB database^4^ as input files for gene set variation analysis (GSVA) (39). Further, pathway enrichment analysis was performed to identify distinct pathways associated with different subtypes.

### 2.10 Differential gene expression analysis and functional annotation of UC subtypes

To assess the reproducibility of the data, PCA was utilized to examine the overall distribution and determine the reproducibility of the data across subtypes. Additionally, a volcano plot was generated for visualizing the DEGs in distant subtypes. Enriched GO terms and KEGG pathways within the differential subtypes were analyzed. Furthermore, the correlation between the top five KEGG pathways and the DEGs in the distinct subtypes was established.

### 2.11 Regulatory network of TFs and miRNAs associated with the key anoikis-DEGs

Transcription factors (TFs) and microRNAs (miRNAs) are pivotal regulators of gene regulations, functioning at the transcriptional and posttranscriptional levels, respectively. To explore TF- and miRNA-mediated gene interactions, the RegNetwork database^5^ was utilized to predict potential upstream miRNAs and TFs (40). Regulatory network of miRNAs and TFs were constructed based on key Anoikis-DEGs using Cytoscape 3.9.0 software.

### 2.12 Statistical analysis

Data analyses were performed utilizing R 4.3.3 and related R packages. GraphPad Prism 9.3.0 (GraphPad Software, Inc., La Jolla, CA, United States) was used for statistical analysis. All data are shown as mean ± standard deviation (SD). Differences between two groups were assessed using the *t*-test. The correlation between the two variables was determined by using the Pearson product–moment correlation coefficient. *p* < 0.05 indicated statistical significance.

## 3 Results

### 3.1 Data processing and identification of DEGs in UC individuals

**Figure 1:**
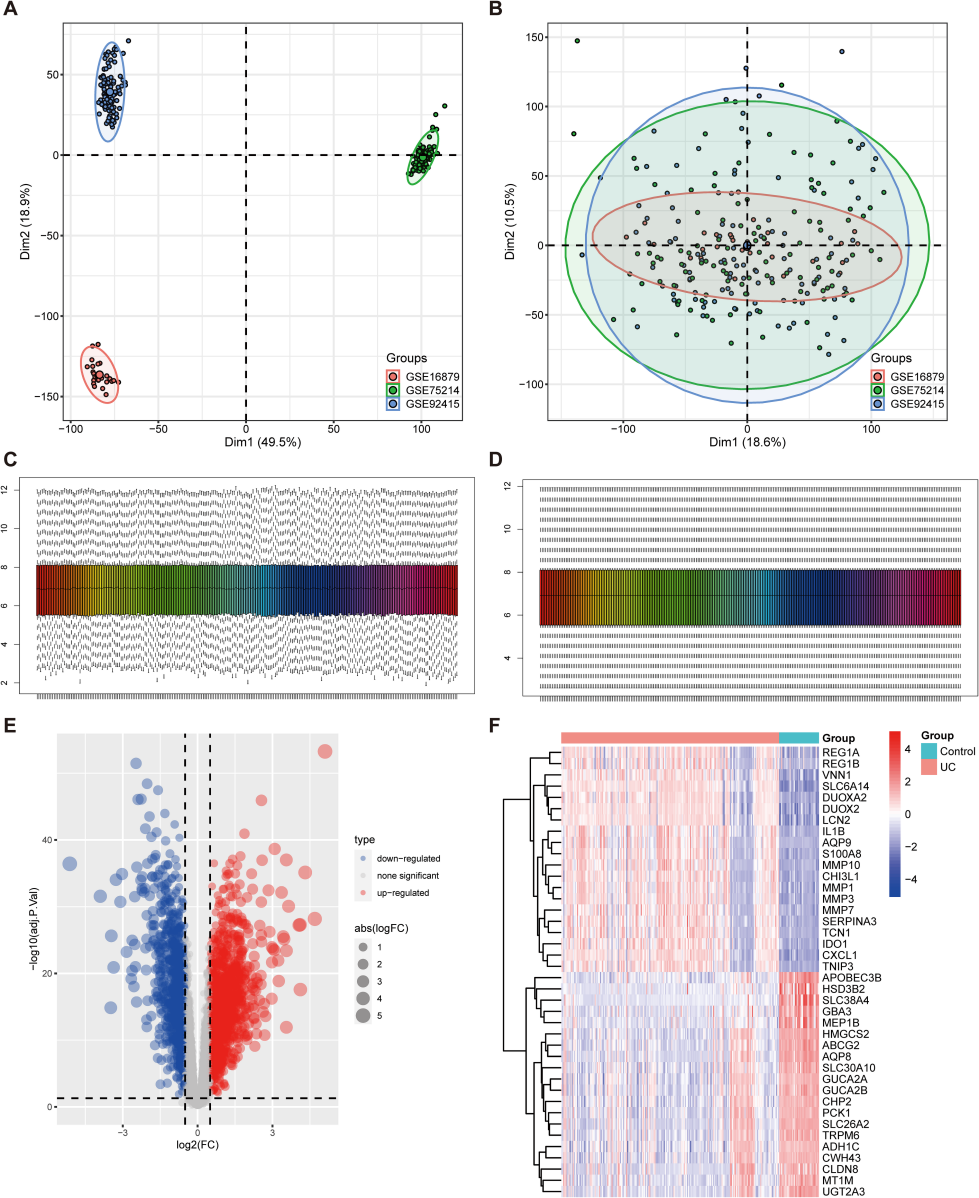
Identification of DEGs associated with UC. **(A,B)** Principal component analysis (PCA) of three datasets before **(A)** and after **(B)** batch effect removal. **(C,D)** Combined data normalized before **(C)** and after **(D)**. **(E)** The volcano plot illustrates the upregulated and downregulated DEGs associated with UC. Horizontal coordinates represent log_2_FC, and vertical coordinates represent -log_10_ (adj.*P*.Val). Red nodes represent upregulated DEGs, blue nodes represent downregulated DEGs, and gray nodes denote genes that do not exhibit significant differential expression. **(F)** Heatmap displays the top 20 upregulated and downregulated DEGs in UC. Red signifies upregulated gene expression, and blue signifies downregulated gene expression.

To examine the biological role of ARGs in UC progression, the expression data of GSE75214, GSE92415, and GSE16879 datasets was firstly integrated. Tissues from different platforms exhibited distinct clustering patterns prior to batch effect removal (Figure 1A). The batch effects were effectively removed from the GEO datasets, resulting in an integrated dataset comprising 208 UC samples and 38 control samples (Figure 1B). Next, the data underwent normalization using the R software package “preprocessCore” (Figures 1C,D). We obtained 2,744 DEGs differentiating UC from normal tissues, comprising 1,577 upregulated and 1,167 downregulated genes, as illustrated by the volcano plot (Figure 1E) and the top 20 genes that have been upregulated and downregulated illustrated in a heatmap (Figure 1F).

### 3.2 Functional enrichment analysis of DEGs

**Figure 2:**
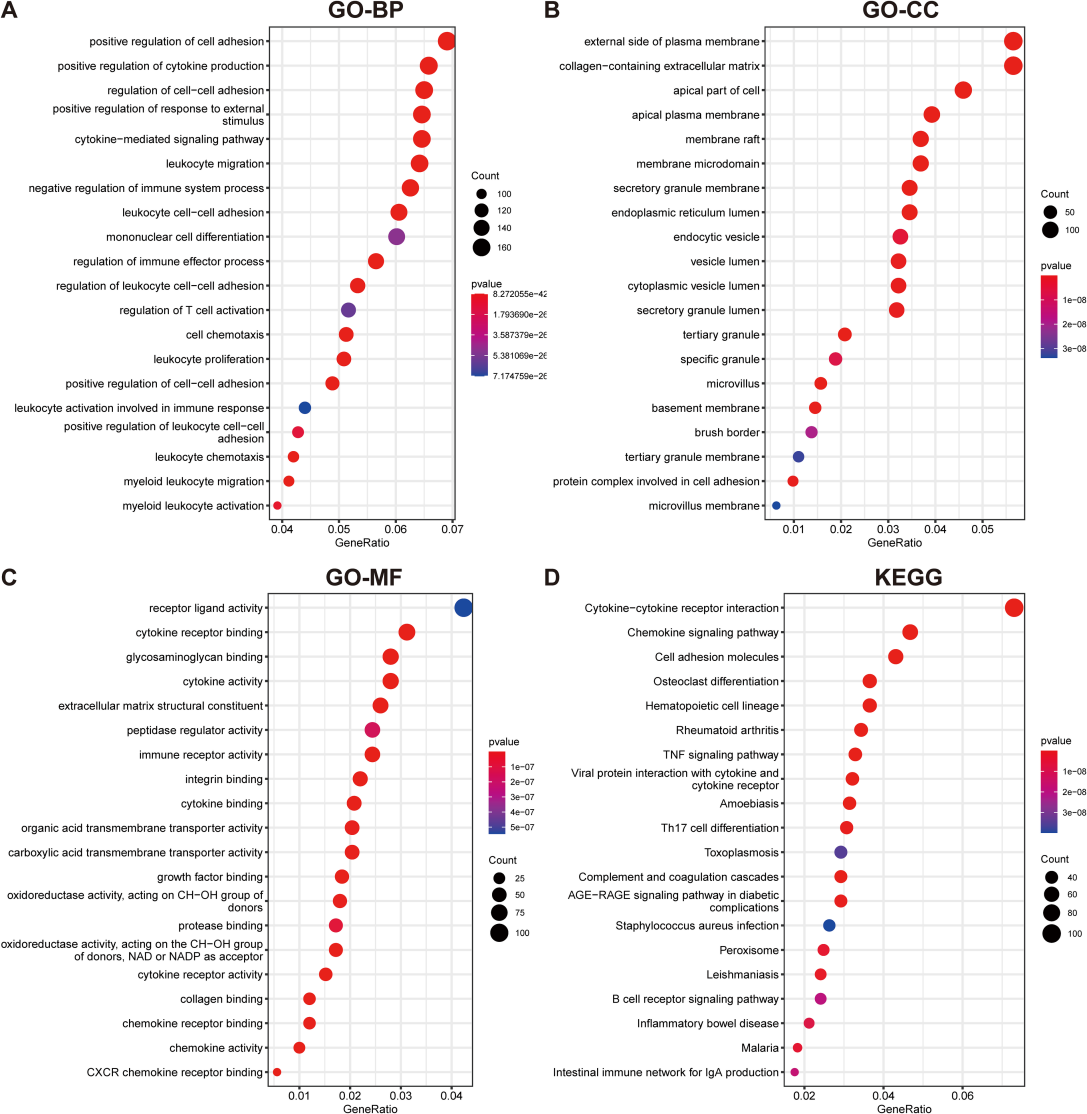
Functional enrichment analyses of DEGs in UC. **(A–C)** GO analysis reveals the top 20 enriched categories for biological processes (BPs) **(A)**, cellular components (CCs) **(B)**, and molecular functions (MFs) **(C)**. **(D)** The top 20 KEGG pathway analysis.

To elucidate the underlying mechanisms and roles of these DEGs in UC, we carried out GO and KEGG pathway analyses. The GO enrichment analysis indicated these genes significantly involved in pathways such as cell adhesion, cytokine production, plasma membrane, and extracellular matrix (Figures 2A–C). Furthermore, the KEGG enrichment analysis demonstrated that the DEGs were predominantly enriched in multiple inflammatory response-related pathways, including the cytokine-cytokine receptor interaction, TNF signaling pathway, Th17 cell differentiation, and B cell receptor signaling pathway (Figure 2D).

### 3.3 Identification of anoikis-DEGs in UC and functional enrichment analysis

**Figure 3:**
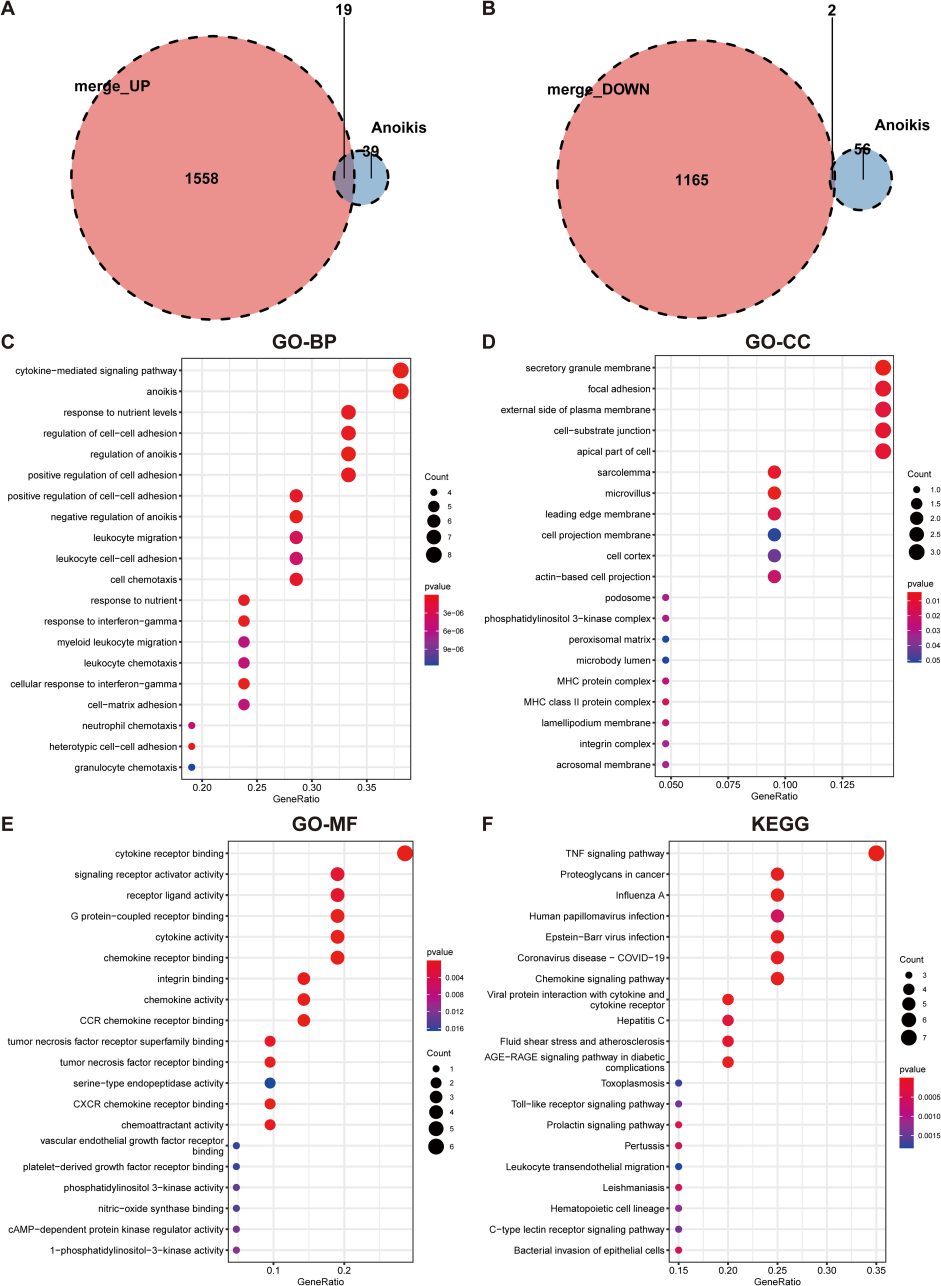
Identification of anoikis-differentially expressed genes in UC and functional enrichment analysis. **(A)** Venn diagram demonstrates the overlapping genes between upregulated DEGs and anoikis-related genes. **(B)** Venn diagram demonstrates the overlapping genes between downregulated DEGs and anoikis-related genes. **(C)** GO-BP enrichment analysis of anoikis-DEGs. **(D)** GO-CC enrichment analysis of anoikis-DEGs. **(E)** GO-MF enrichment analysis of anoikis-DEGs. **(F)** KEGG pathway enrichment analysis of anoikis-DEGs.

To ascertain the role of ARGs in UC, the overlap between anoikis genes and DEGs in UC was initially identified. By intersecting the upregulated and downregulated DEGs with anoikis, 19 upregulated anoikis-DEGs (*HLA-DMA*, *CFB*, *CD44*, *ITGA5*, *VCAM1*, *STAT1*, *PIK3R3*, *CCL20*, *NOS2*, *IRF1*, *CX3CL1*, *SIK1*, *CXCL10*, *CAV1*, *CEACAM6*, *MMP2*, *MX1*, *LTA*, and *SNAI2*) and two downregulated genes (*PDK4* and *DAPK2*) in UC were identified (Figures 3A,B). Subsequently, we conducted GO and KEGG enrichment analyses utilizing the 21 anoikis-DEGs. The GO term analysis highlighted that anoikis-DEGs exhibited enrichment in BPs, including cytokine-mediated signaling pathway, anoikis, regulation of cell–cell adhesion, and regulation of anoikis (Figure 3C). For CCs, enrichment was observed in the secretory granule membrane, focal adhesion, external side of the plasma membrane, and cell-substrate junction (Figure 3D). For MFs, the enriched functions included cytokine receptor binding, signaling receptor activator activity, receptor ligand activity, and G protein-coupled receptor binding (Figure 3E). KEGG analysis demonstrated anoikis-DEGs exhibited enrichment in the TNF signaling pathway, Chemokine signaling pathway, and Toll-like receptor signaling pathway (Figure 3F).

### 3.4 Differential gene expression analysis of ARGs between UC and healthy individuals

**Figure 4:**
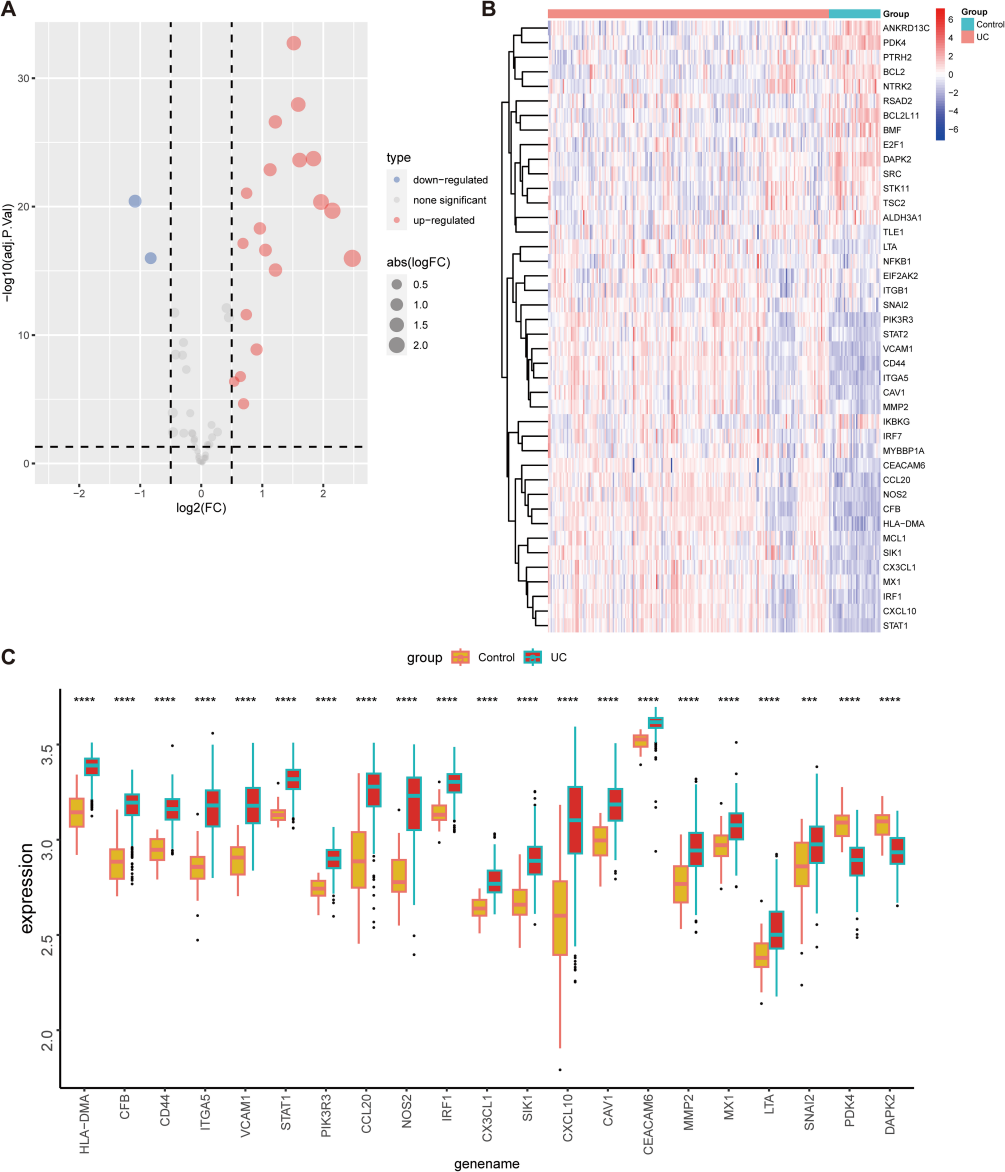
Expression of anoikis-related genes in UC and healthy individuals. **(A)** Volcano map of anoikis-related genes between individuals with UC and healthy controls. **(B)** Heatmap depicts the expression profiles of anoikis-related genes between individuals with UC and healthy controls. **(C)** The bar plot demonstrates the differential expression of anoikis-related genes between UC patients and healthy controls in the merged dataset. ***p < 0.001, ****p < 0.0001.

To evaluate the expression profiles of ARGs in UC and healthy individuals, we performed differential gene expression analysis. The volcano plot in Figure 4A illustrated the upregulation of 19 anoikis-DEGs and the downregulation of two genes in UC patients. Moreover, Figure 4B presented a heatmap displaying the differential expression of ARGs between UC groups and healthy control. Next, we compared the expression of these 21 anoikis-DEGs between UC patients and healthy individuals. Specifically, *PDK4* and *DAPK2* demonstrated relatively low expression in UC. In contrast, the other 19 genes (*CEACAM6*, *CFB*, *HLA-DMA*, *CX3CL1*, etc.) showed significant elevation in UC compared with normal samples (Figure 4C). Moreover, a PPI network was created to explore the relationship between anoikis-DEGs (Supplementary Figure 1). Among these, *CXCL10*, *VCAM1*, *CCL20*, *STAT1*, and *CX3CL1* highlighted their interaction with other anoikis-related proteins. The PPI network highlights critical immune and inflammatory pathways, particularly involving *VCAM1* and *CXCL10*, providing insights into the molecular mechanisms that may potentially contribute to UC pathogenesis.

### 3.5 Key anoikis-DEGs identification through machine-learning

**Figure 5:**
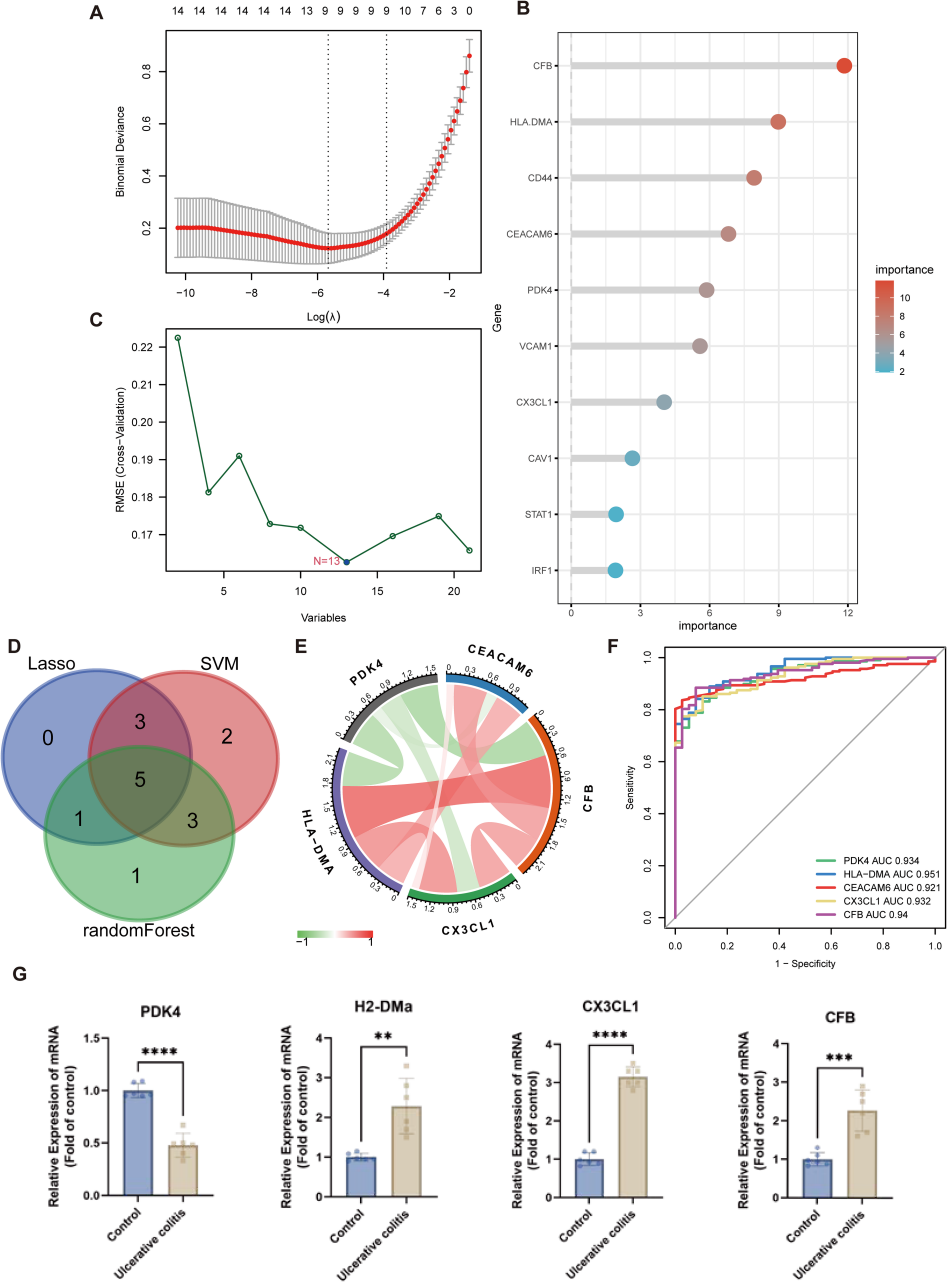
Machine learning algorithms for identifying the key anoikis-DEGs. **(A)** LASSO Cox regression analysis. Vertical dashed lines are plotted at the optimal lambda value. **(B)** Random forest algorithm ranks the importance of anoikis-DEGs. **(C)** SVM-RFE algorithm for signature gene selection. **(D)** Venn diagram displays the overlapping genes between LASSO Cox regression, SVM-RFE algorithms, and random forest method. **(E)** The chord diagram shows the correlations between the five key anoikis-DEGs. **(F)** ROC curves of the five key anoikis-DEGs display their diagnostic value. **(G)** qPCR analysis validation of the key anoikis-DEGs in the mouse model of UC (*n* = 6 for each group). **p < 0.01, ***p < 0.001, ****p < 0.0001.

Three machine-learning algorithms were employed to identify key anoikis-DEGs as characteristic biomarkers of UC from a set of 21 anoikis-DEGs. The LASSO Cox regression analysis demonstrated that nine genes were associated with UC, namely *HLA-DMA*, *CFB*, *CCL20*, *CX3CL1*, *SIK1*, *CAV1*, *CEACAM6*, *PDK4*, and *DAPK2* (Figure 5A). The RF algorithm ranked the importance scores of signature genes and identified 10 genes, including *CFB*, *HLA-DMA*, *CD44*, *CEACAM6*, *PDK4*, *VCAM1*, *CX3CL1*, *CAV1*, *STAT1*, and *IRF1* (Figure 5B). In the case of SVM outcomes, 13 signature genes were identified, namely *ITGA5*, *HLA-DMA*, *CX3CL1*, *CD44*, *PDK4*, *VCAM1*, *STAT1*, *CFB*, *SIK1*, *CEACAM6*, *DAPK2*, *PIK3R3*, and *CCL20* (Figure 5C). Subsequently, after intersecting of the three machine learning results, five key anoikis-DEGs, namely *PDK4*, *HLA-DMA*, *CEACAM6*, *CX3CL1*, and *CFB* were identified (Figure 5D). Additionally, a correlation analysis was conducted on these five key anoikis-DEGs. Except for *PDK4*, which exhibited a negative correlation with the other four genes, all the other genes showed positive correlations with each other (Figure 5E). Subsequently, ROC analysis was carried out to assess the predictive value of these genes. ROC curves are commonly used to assess the performance of risk prediction models and the AUC quantifies this discriminative ability, with higher AUC values indicating better model performance. Moreover, an AUC curve that closely follows the top-left corner of the plot indicates higher accuracy, reflecting a model with strong predictive capability. In Figure 5F, the results indicated that each of the five genes exhibited relatively high predictive values (AUC values ≥0.921). Thus, these genes can serve as biomarkers for early diagnosis, disease monitoring, or evaluation the therapeutic effects. By conducting further research on the functions and mechanisms of these genes, we can gain a deeper understanding of the pathophysiological process of UC and provide clues for the development of new treatment approaches. By using the DSS-induced mouse colitis model, we aimed to substantiate the critical role of key anoikis genes in active UC. Because the *CEACAM6* gene is not present in rodents, and *H2-DMa* is orthologous to human *HLA-DMA*, we ultimately analyzed the expression of four key anoikis genes: *PDK4*, *H2-DMa*, *CX3CL1*, and *CFB* in UC and normal samples (Figure 5G). The qPCR results showed an increase in *H2-DMa*, *CX3CL1*, and *CFB* in the DSS group, while *PDK4* was decreased, consistent with the results from UC patients in Figure 4C.

### 3.6 The immune infiltration characteristics in UC samples and the association between key anoikis-DEGs and infiltrating immune cells

**Figure 6:**
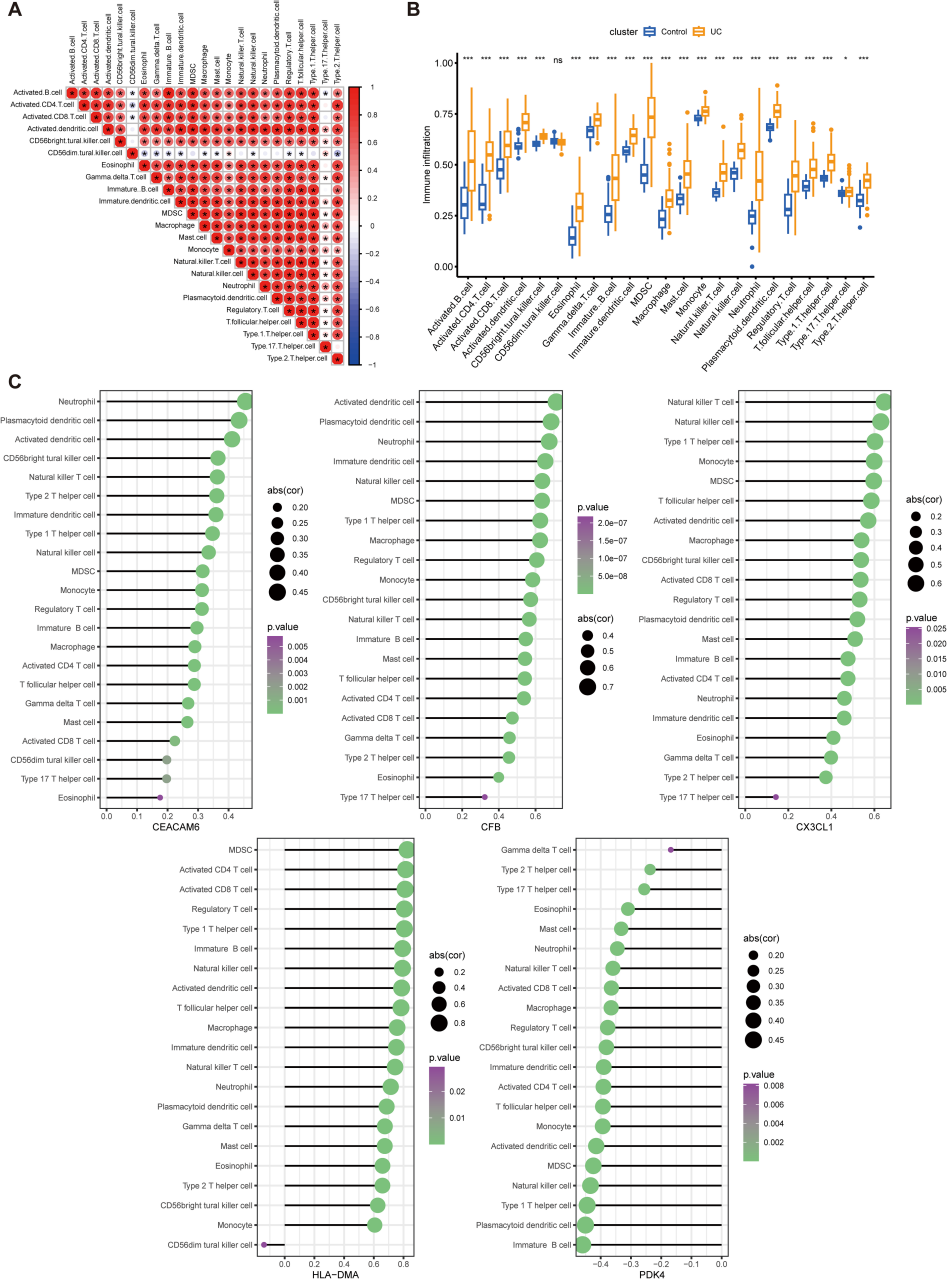
Analysis of immune infiltration characteristics in UC samples and the correlation between key Anoikis-DEGs and infiltrating immune cells. **(A)** Correlation analysis among 23 immune cells. The size and color of the circles indicate the correlation coefficient, with blue and red colors representing positive and negative correlation, respectively. The circle size is proportional to the correlation coefficient value. **(B)** Boxplots illustrate the variations in infiltrated immune cells between UC and healthy individuals. **(C)** Lollipop charts present the correlations between the five key anoikis-DEGs genes and immune cell infiltration. The size and color of the circles demonstrate the correlation coefficient, with purple and green colors representing the *p*-value and the circle size indicating the correlation coefficient value. *p < 0.05, **p < 0.01, ***p < 0.001, ****p < 0.0001, ns: no significance.

To determine if UC patients exhibited altered immune system activity, an assessment of the infiltration and correlations of 23 distinct immune cell subtypes in UC was conducted. Figure 6A depicts multiple correlations among the infiltrating immune cells in UC. A significant synergistic effect was observed between activated B cell, activated CD4 cell, activated CD8 cell, activated dendritic cell, eosinophil, gamma delta T cell, macrophage, mast cell, monocyte, natural killer T (NKT) cell, natural killer (NK) cell, neutrophil, plasmacytoid dendritic cell (pDC), regulatory T cell (Treg), type 1 T helper (Th1) cell, and type 2 T helper (Th2) cell. Moreover, activated dendritic cell, macrophage, monocyte, neutrophil, pDC, and Th1 cell exhibited synergism with Th17 cell. In contrast, a competitive effect was observed between CD56^dim^ NK cells and most other immune cells. Subsequently, the immune infiltration analysis was performed and compared between the UC and normal groups. A significant elevation of 22 immune cell subtypes was observed in the immune cell infiltration within UC group (Figure 6B). These immune cell types included activated B cell, activated CD4 cell, activated CD8 cell, activated dendritic cell, CD56^bright^ NK cell, eosinophil, gamma delta T cell, immature B cell, immature dendritic cell, MDSC, macrophage, mast cell, monocyte, NKT cell, NK cell, neutrophil, plasmacytoid dendritic cell, regulatory T cell, T follicular helper cell, Th1 cell, Th17 cell, and Th2 cell. These cell types were remarkably abundant in the UC samples. Furthermore, lollipop charts present the associations between the five key anoikis-DEGs and immune cell infiltration (Figure 6C). *CEACAM6*, *CFB*, *CX3CL1*, and *HLA-DMA* exhibited positive associations with most of the immune cells. In contrast, *PDK4* demonstrated a negative correlation with all immune cells.

### 3.7 Correlation of five key anoikis-DEGs with all UC genes and GSEA analysis

The correlations between five key anoikis-DEGs and all merged genes were investigated, and the top 50 positively correlated genes were visualized in Supplementary Figure 2. Moreover, the Reactome pathway enrichment analyses of the five key anoikis-DEGs were conducted utilizing GSEA to identify significant biological signatures, and the top 20 pathways were presented (Supplementary Figure 3). For each key anoikis-DEGs, the top 20 pathways had the same adjusted *p*-value, which indicates that, statistically, they are equally important. In UC, the Reactome pathways most positively associated with *CEACAM6* included the ER-phagosome pathway, antigen processing-cross presentation, asparagine N-linked glycosylation, and transport to the Golgi and subsequent modification. The Reactome pathways most positively associated with *CFB* were immunoregulatory interactions between a lymphoid and a non-lymphoid cell, interferon alpha/beta signaling, integrin cell surface interactions, and antigen processing-cross presentation. The top positively affected Reactome pathways of *CX3CL1* included immunoregulatory interactions between lymphoid and non-lymphoid cells, interleukin-4 and interleukin-13 signaling, interferon alpha/beta signaling, and integrin cell surface interactions. The significantly enriched pathways of *HLA-DMA* included immunoregulatory interactions between a lymphoid and a non-lymphoid cell, cytokine signaling in immune system, signaling by interleukins, and interleukin-4 and interleukin-13 signaling. Most pathways positively related to *PDK4* included the citric acid (TCA) cycle and respiratory electron transport, peroxisomal lipid metabolism, pyruvate metabolism and TCA cycle, and other pathways.

### 3.8 Construction of anoikis-related subtypes and gene expression profiles in UC

**Figure 7:**
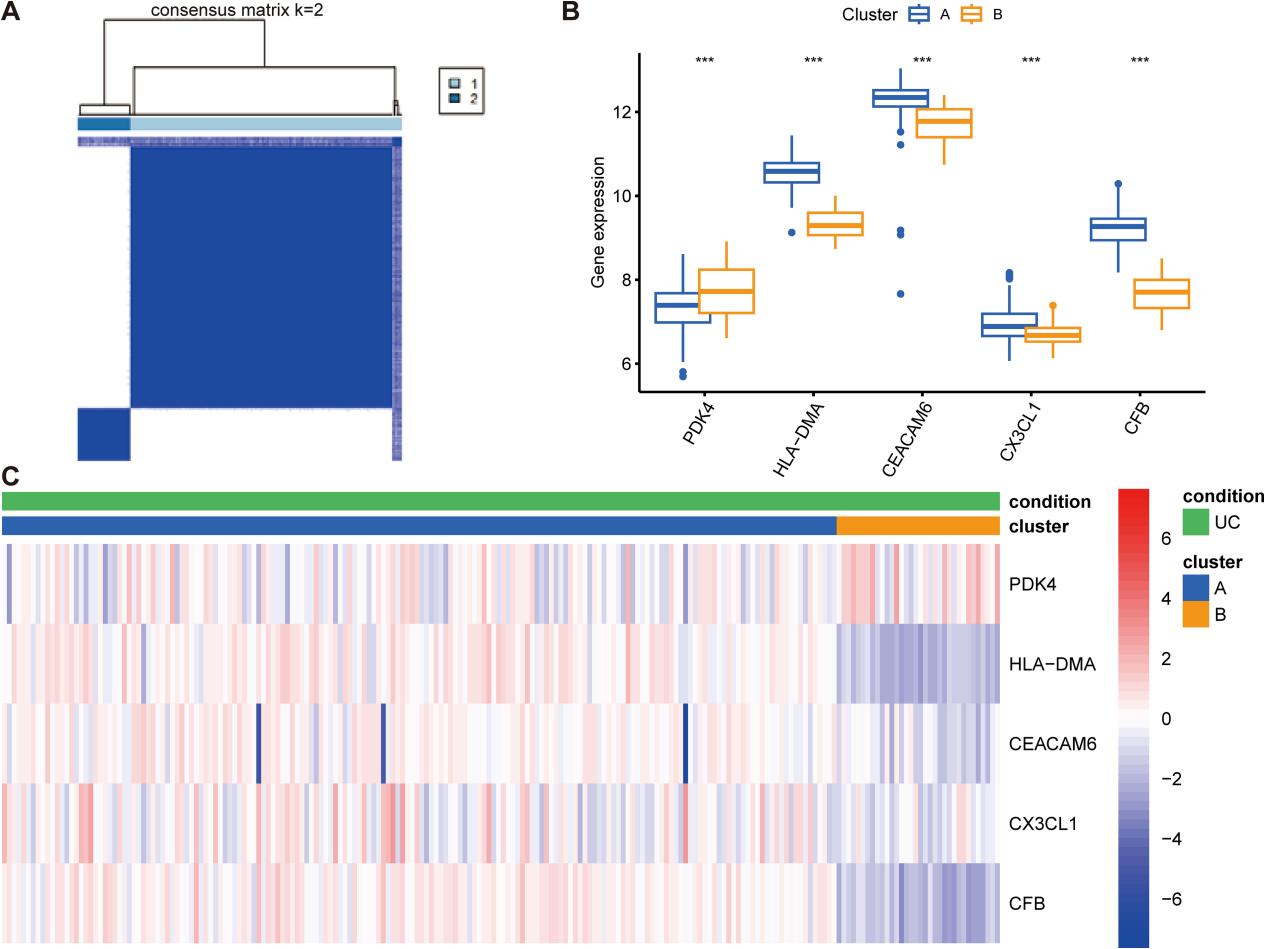
Construction of anoikis-related subtypes and gene expression profiles in UC. **(A)** Unsupervised clustering analysis at k = 2. The consensus matrix showed the best performance, indicating that the optimal number of clusters was 2. **(B)** Differences in the expression of the five key anoikis-DEGs between the two subgroups. **(C)** Heatmap of the expression of five key anoikis-DEGs and clinical profiles of subgroups in UC. Red and blue colors demonstrate positive and negative associations, respectively. ***p < 0.001.

The 208 UC samples were classified as per the expression profiles of the five key anoikis DEGs utilizing an unsupervised clustering analysis. Various values of k were tested, varying from 2 to 9, and it was found that when k = 2, the consensus matrices showed the best performance, indicating that the optimal number of clusters was 2 (Figure 7A). Differences in the five key anoikis-DEGs expression between the two groups were observed. Specifically, higher expression of *HLA-DMA*, *CEACAM6*, *CX3CL1*, and *CFB* and lower expression of *PDK4* were observed in cluster A (Figure 7B). Furthermore, a heatmap demonstrated the relationships among the clinical features, expression of the five key anoikis-DEGs, and subtypes (Figure 7C).

### 3.9 Functional enrichment analysis between UC different subtypes

**Figure 8:**
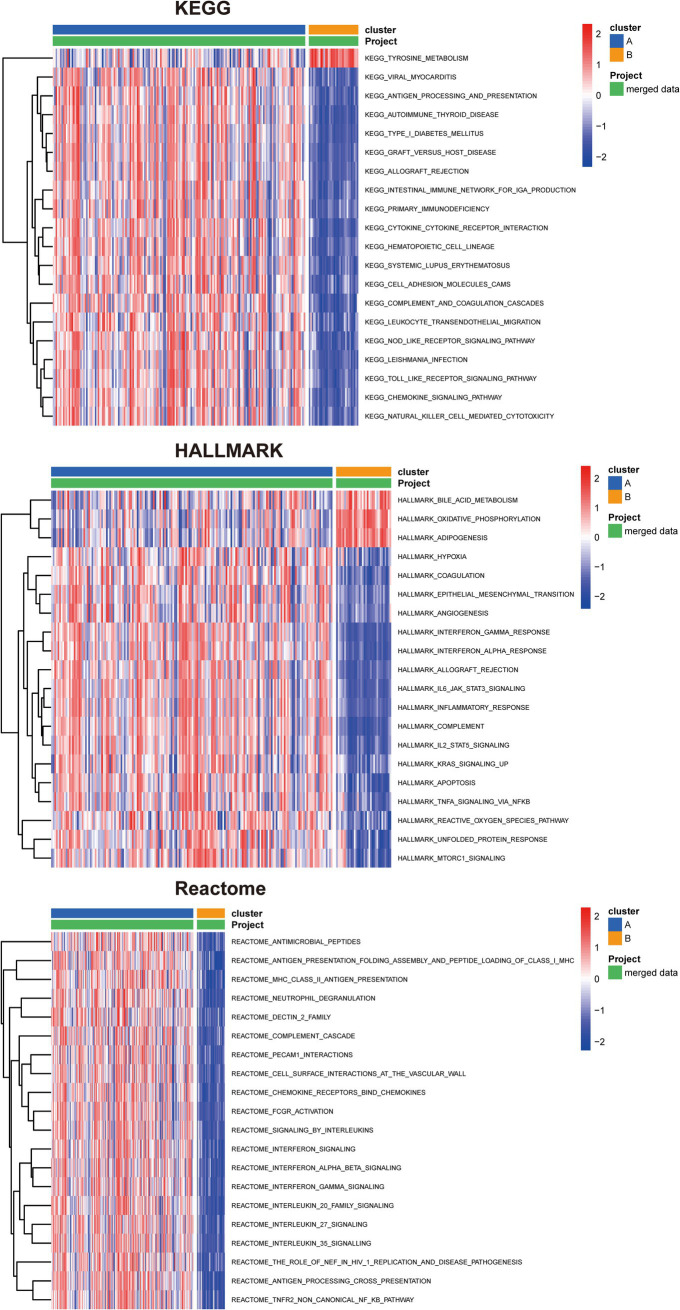
Identification of biological functional characteristics in different clusters of UC. The heatmap shows the top 20 significant KEGG, HALLMARK, and Reactome pathways between two clusters of UC separately.

We subsequently conducted pathway enrichment analysis to investigate whether distinct pathways were perturbed in patients with different subtypes. The KEGG, Hallmark, and Reactome pathway analyses were performed to uncover the underlying pathways that differentiate cluster A from cluster B (Figure 8). Our findings revealed that these subtypes enriched in distinctive KEGG pathways. Cluster A displayed enhanced activation in antigen processing and presentation, intestinal immune network for IgA production, cytokine-cytokine receptor interaction, cell adhesion molecules, NOD-like receptor signaling pathway, Toll-like receptor signaling pathway, chemokine signaling pathway, and NK cell-mediated cytotoxicity. Furthermore, specific enrichment of tyrosine metabolism was observed in cluster B. In cluster A, Hallmark gene sets associated with the following pathways were upregulated: IL-6/JAK/STAT3 signaling, IL2/STAT5 signaling, apoptosis, TNF-*α* signaling *via* NF-κB, reactive oxygen species pathway, and mTORC1 signaling. In contrast, bile acid metabolism, oxidative phosphorylation, and adipogenesis were upregulated in cluster B. Reactome analyses indicated that antigen presentation: folding assembly and peptide loading of class I MHC, chemokine receptors bind chemokines, interferon signaling, and TNFR2 non-canonical NF-κb pathway were activated in cluster A. Taken together, these results suggest that different UC subtypes are regulated by distinct immune pathways.

### 3.10 Differential gene expression analysis and functional enrichment between UC subtypes

**Figure 9:**
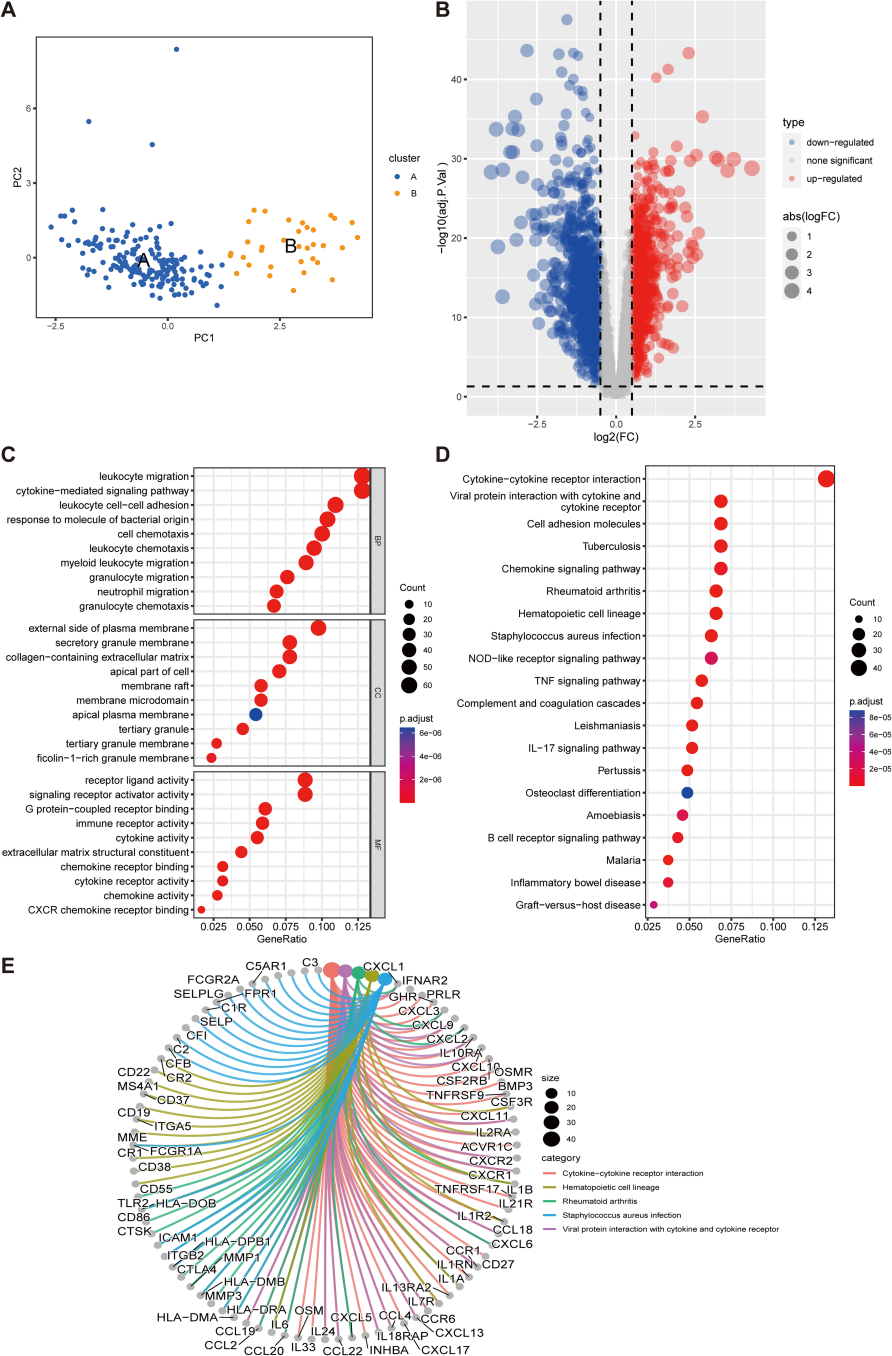
Differential gene expression analysis and functional annotation of UC subtypes. **(A)** Principal component analysis (PCA) to visually represent the distribution of the two subtypes. **(B)** Volcano diagram of 579 differentially expressed genes of the two subtypes, with red, blue, and gray dots representing upregulated, downregulated, and no significant difference, respectively. **(C,D)** GO and KEGG enrichment analyses. **(E)** Relationship between the top five KEGG pathways and differential genes.

The distinct distribution of subsets was visualized through PCA to account for the differences between cluster A and B subtypes (Figure 9A). Moreover, a differential gene analysis comprising 579 genes of the two subtypes was conducted, presented as a volcano plot (Figure 9B). The GO enrichment analysis highlighted that the DEGs were primarily linked to pathways such as cytokine-mediated signaling pathway, leukocyte cell–cell adhesion, cell chemotaxis in BP, external side of plasma membrane, secretory granule membrane, collagen-containing extracellular matrix in CC, receptor ligand activity, signaling receptor activator activity, immune receptor activity, cytokine activity, and cytokine receptor activity in MF (Figure 9C). KEGG analysis indicated enrichment in cytokine-cytokine receptor interaction, cell adhesion molecules, chemokine signaling pathway, NOD-like receptor signaling pathway, TNF signaling pathway, IL-17 signaling pathway, and inflammatory bowel disease (Figure 9D). Moreover, Figure 9E illustrated the relationship between the top five KEGG pathways and differential genes.

### 3.11 Regulatory network of TFs and miRNAs associated with the key anoikis-DEGs

To examine the potential upstream regulators of the five key genes, the RegNetwork database was utilized to extract relationships between upstream miRNAs and TFs likely to bind to these five key genes (Supplementary Figure 4). The results revealed a multitude of miRNAs and TFs implicated in the modulation of these diagnostic genes. TP53, RARB, RXRB, and CTCF were identified as regulators of multiple genes. Especially, CTCF is critically involved in regulating *CFB*, *CXCL3*, *HLA-DMA*, and *CEACAM6*.

## 4 Discussion

UC has emerged as a global health concern due to its high prevalence in developed nations and a significant rise in occurrence in developing countries (3, 41). Pathogenesis of UC is associated with disrupted intestinal barriers, imbalance of the gut microbiome, and subsequent dysregulated mucosal immune responses to gut commensal bacteria. At present, the primary treatment for moderate and severe UC is anti-TNF therapy, leading to a substantial improvement in treatment outcomes (41). Nevertheless, many patients remain unresponsive to anti-TNF therapy and develop colitis-associated colorectal dysplasia or cancer, warranting restorative proctocolectomy. Therefore, thoroughly exploring the potential mechanisms of UC and discovering new biomarkers that can aid in the development of novel UC treatment strategies are imperative. Considerable efforts from researchers have been directed toward investigating novel diagnostic techniques and therapeutic approaches with the aim of improving early diagnosis and treatment of UC. Anoikis fundamentally represents a form of programmed cell death that shares similarities with classical apoptosis (9). This process can manifest through two distinct pathways akin to classic apoptosis. One is the extrinsic pathway, initiated by death receptors located on the cell surface, while the other is the intrinsic pathway, which involves mitochondria-mediated mechanisms (Supplementary Figure 5). Nevertheless, the underlying mechanisms through which anoikis regulates UC remain in need of further exploration.

We thoroughly investigated the differential gene profiles between 208 UC samples and 38 control samples obtained from the GEO datasets. Moreover, three machine learning algorithms, including LASSO, RF and SVM, were employed to examine the involvement of ARGs in UC. The 2,744 DEGs between UC and healthy tissues exhibited enrichment in cytokine-cytokine receptor interaction, TNF signaling pathway, and Th17 cell differentiation pathways (Figure 2D). Furthermore, 21 dysfunctional ARGs were identified among UC individuals. This discovery underscores the plausible significance of anoikis in the progression of UC. Among them, *PDK4* and *DAPK2* exhibited relatively low expression levels in UC. In contrast, the other 19 genes, such as *CEACAM6*, *CFB*, *HLA-DMA*, *CX3CL1*, and others were notably enhanced in UC compared to normal samples (Figure 4C).

Recently, utilization of machine learning in diagnosing UC through the screening of key genes and immune cells has been widely used. This is attributed to its superior predictive performance, reduced error rates, and enhanced reliability (42, 43). In this study, five key anoikis-DEGs, *PDK4*, *HLA-DMA*, *CEACAM6*, *CX3CL1*, and *CFB* were identified as characteristic biomarkers of UC using LASSO, RF, and SVM algorithms (Figure 5). These five signature genes displayed strong diagnostic values (all AUC values ≥0.921). Therefore, these five specific genes possess predictive value for the occurrence of UC.

Several researches have shown the participation of some of these key diagnostic genes in the pathogenesis of UC. For example, pyruvate dehydrogenase kinase isozyme 4 (PDK4) is crucial for regulating both glucose and fatty acid metabolism as well as maintaining homeostasis. Cyclosporine A is reported to facilitate neutrophil glycolysis and the TCA cycle by suppressing Sirtuin 6 (SIRT6) and promoting PDK4, ultimately alleviating clinical symptoms in severe UC (44). Our results demonstrate a negative association between PDK4 and neutrophil count in UC (Figure 6). The carcinoembryonic antigen-related adhesion molecules (CEACAM) are expressed on various cell types, comprising epithelial cells, neutrophils, and T cells (45). It is implicated in several processes, including proliferation, cell adhesion, differentiation, and tumor suppression. CEACAM6 is expressed on granulocytes and monocytes, and its expression is highly enhanced in individuals with Crohn’s disease (CD), another chronic intestinal inflammatory disorder (46–48). A transgenic CEABAC10 mouse model infected with adherent-invasive *Escherichia coli* (AIEC) and expressing human CEACAM6 demonstrated increased proinflammatory cytokine IL-6 and IL-17 levels, reduced anti-inflammatory cytokine IL-10 levels, and histopathological damage to the gut mucosa (46). Meanwhile, blocking CEACAM6 with monoclonal antibodies reduced AIEC colonization and the inflammatory response. This suggests that CEACAM6 could be a potential treatment target for CD. The chemokine fractalkine (CX3CL1) is synthesized as a type I transmembrane protein (49). Vascular endothelial cells express CX3CL1, which is upregulated in response to proinflammatory cytokine stimulation. The CX3CR1, a receptor for fractalkine, is highly expressed on monocytes/macrophages, cytotoxic lymphocytes, and dendritic cells. The CX3CL1-CX3CR1 pathway is critically involved in gastrointestinal mucosal immunity. CX3CL1 is significantly upregulated in the mucosal epithelia and vascular endothelium of patients with active CD (50). Moreover, in a transfer IBD model, the anti-CX3CL1 monoclonal antibodies effectively mitigated the reduction in body weight, alleviated diarrhea, and reduced colon thickness. The gene Complement Factor B (CFB) encodes a secreted protein that plays a role in activating the alternative pathway of complement and is expressed primarily by the liver and mononuclear phagocytes (51, 52). The complement system is essential for pathogen lysis, opsonization, inflammation, and immune clearance. Previous research revealed that both CFB mRNA and protein exhibited elevated expression levels in the colonic mucosa of UC patients, indicating its involvement in the inappropriate activation of complement system (53). This activation contributes to chronic inflammation, resulting in active UC. Hence, the key anoikis-DEGs, *PDK4*, *CEACAM6*, *CX3CL1*, and *CFB* are closely related to immune cell infiltration and inflammatory pathways.

The primary pathological damage in UC arises from dysregulated immune responses, which leads to the production of inflammatory cytokines and the activation of immune cell signaling. These key anoikis genes are deeply involved in inflammatory processes and associated with the pathology of UC, particularly CEACAM6 and CX3CL1. In the future, therapies targeting key anoikis-DEGs, especially blocking CEACAM6 and CX3CL1-CX3CR1 pathway with monoclonal antibodies could effectively improve UC pathology by ameliorating the inflammatory response. Additionally, the current biomarkers for UC included serum anti-αvβ6 antibodies and serum oncostatin M for diagnosis, fecal calprotectin and serum trefoil for assessing disease activity, as well as whole blood transcriptomic panels and CLEC5A/CDH2 ratio for determining the need for escalated treatment (5). The diagnostic and prognostic accuracy is often limited by factors such as variability in disease stage or response to treatment. However, the ARGs have considerable advantages as biomarkers for UC. CEACAM6 has been associated with the activation of inflammatory pathways and immune cell migration (45). Elevated levels of CEACAM6 have been associated with chronic inflammation and could serve as a more specific marker for immune dysregulation in UC compared to serum oncostatin M. CX3CL1, as a chemokine involved in immune cell trafficking, plays a crucial role in gastrointestinal mucosal immunity (50). It is significantly upregulated in active UC and could serve as a novel marker for UC and immune response regulation, complementing biomarkers like fecal calprotectin. CFB has been identified as a key component in the complement system (51, 52). Elevated expression of CFB in the colonic mucosa of UC patients suggests its involvement in immune activation and could serve as an early indicator of UC onset or exacerbation, in contrast to the more general serum trefoil factors used for disease activity assessment. PDK4 plays a role in metabolic regulation and cell survival (44). Its downregulation is associated with inflammation and immune cell infiltration, suggesting that it may serve as a marker for UC progression and tissue damage, potentially complementing whole blood transcriptomic panels used for determining UC severity. By focusing on these key ARGs, we highlighted their potential roles as specific and reliable biomarkers for UC, particularly in identifying disease activity, immune dysregulation, and the need for targeted therapeutic interventions. These ARGs can provide a more targeted approach to managing UC, improving diagnostic accuracy, and guiding clinical decisions. Of note, DSS-induced colitis mouse model was constructed to demonstrate that the expression of key anoikis genes, namely *H2-DMa*, *CX3CL1*, and *CFB* were significantly upregulated, while *PDK4* was evidently downregulated. These results are in accordance with the previous results. However, additional researches are necessary to reveal the underlying mechanisms of *HLA-DMA* in UC. In summary, the studies involving these key anoikis-DEGs validate the reliability of our screening outcomes.

UC is characterized by a persistent chronic inflammatory triggered by an overactive immune response against gut bacteria or dietary antigens (3). This abnormal immune response involves both innate and adaptive immune cells, playing pivotal roles in the development and escalation of UC. Among these, innate immune cells such as dendritic cells, neutrophils, and macrophages have a substantial effect in the pathogenesis of UC. Additionally, innate lymphoid cells, which have garnered increased attention recently, also contribute to the disease. Simultaneously, the adaptive immune response mechanisms encompass various types of cells, including cytotoxic T lymphocytes, Tregs, and different subsets of helper T lymphocytes (Th), such as Th1, Th2, Th9, Th17, and Th22 (7). A recent study revealed increased abundances of IL1B^+^ macrophages and monocytes, IL17A^+^ CD161^+^ effector memory T cells, and IL17A^+^ regulatory T cells in colonic mucosa samples from UC patients (54). Additionally, a significant accumulation of CD11b^+^ B cells has been observed in the intestinal lamina propria and Peyer’s patches in both mouse models with colitis induced by dextran sulfate sodium and individuals with UC. The adoptive transfer of CD11b^+^ B cells successfully ameliorated colitis symptoms and showed therapeutic benefits (55). In this research, we analyzed the immune cell infiltration and found higher enrichment of activated dendritic cells, macrophages, neutrophils, multiple T cells (activated CD4, activated CD8, gamma delta T, NKT, Treg, Th1, Th2, and Th17 cells), and B cells (activated and immature B cells) in UC compared to normal group. Further, our results showed that among the key anoikis-DEGs, *CEACAM6*, *CFB*, *CX3CL1*, and *HLA-DMA* exhibited a positive association with immune cells, while *PDK4* exhibited a negative association with immune cells (Figure 6C). These findings deepen our understanding of immune dysregulation and immune cell involvement in UC.

The enrichment analysis revealed the involvement of five key anoikis genes in immunoregulatory interactions between lymphoid and non-lymphoid cells, interferon alpha/beta signaling, interleukin-4 and interleukin-13 signaling, and antigen processing-cross presentation, among others (Supplementary Figure 3). Using the clustering algorithm, patients were classified into two clusters according to the five key anoikis genes (Figure 7A). *HLA-DMA*, *CEACAM6*, *CX3CL1*, and *CFB* exhibited high expression levels in cluster A, whereas *PDK4* displayed relatively low expression in cluster A (Figure 7B). Moreover, the relationship among the clinical profiles, five key anoikis gene expression, and subtypes was visualized using a heatmap (Figure 7C). To better understand the differences between UC subtypes, the underlying pathways were analyzed, revealing that the two subtypes displayed distinct pathways (Figure 8). Additionally, the interaction network among these characteristic genes, miRNA, and TFs were explored (Supplementary Figure 4), providing insights into the upstream signaling pathways for our subsequent research. In the future, we will persist in exploring the potential mechanisms underlying UC by conducting molecular biology experiments.

Nevertheless, this research has some limitations. Initially, this research relied on a publicly available dataset, and it is crucial to emphasize that these findings generated using bioinformatics techniques require further validation to ensure their reliability. Furthermore, it is essential to highlight that the expression levels of the above genes among individuals from distinct regions or racial backgrounds remain unclear. More *in vitro* and *in vivo* studies are needed to fill these knowledge gaps and understand how these crucial anoikis genes are linked to different immune signaling pathways in UC. These experiments will provide deeper insights into the potential mechanisms underlying the correlation of *PDK4, HLA-DMA, CEACAM6, CX3CL1*, and *CFB* with the infiltration of immune cells in UC, helping to establish a more comprehensive understanding of the disease.

## 5 Conclusion

This research offers a thorough examination of the involvement of ARGs in UC, marking the first instance of revealing the expression profiles of ARGs in UC and their association with immune cells infiltration. Machine learning algorithms and unsupervised clustering analysis based on ARGs were employed to identify five signature genes: *PDK4*, *HLA-DMA*, *CEACAM6*, *CX3CL1*, and *CFB*. These genes are critically involved in immune cell infiltration and immune signaling pathways, showing a considerable diagnostic value. The results offer insights into the classification of UC patients into two clusters, each regulated by distinct pathways. Our findings have the potential to be a valuable reference, offering deeper insights into the underlying mechanisms of anoikis in UC. They could serve as a foundation for the development of innovative strategies in drug screening, personalized treatment, and immunotherapy for individuals with UC.

## Data Availability

The original contributions presented in the study are included in the article/Supplementary material, further inquiries can be directed to the corresponding author/s.

## Footnotes

^1^ https://www.ncbi.nlm.nih.gov/geo/

^2^ https://www.gsea-msigdb.org/gsea

^3^ https://pga.mgh.harvard.edu/primerbank/

^4^ https://www.gsea-msigdb.org/gsea/msigdb/

^5^ https://regnetworkweb.org/
