# Supplementary figures and images for: Comprehensive analysis of anoikis-related gene signature in ulcerative colitis using machine learning algorithms
Source: Front Med (Lausanne). 2025 Mar 6;12:1498864. doi: 10.3389/fmed.2025.1498864 (PMC11922952; doi:10.3389/fmed.2025.1498864)

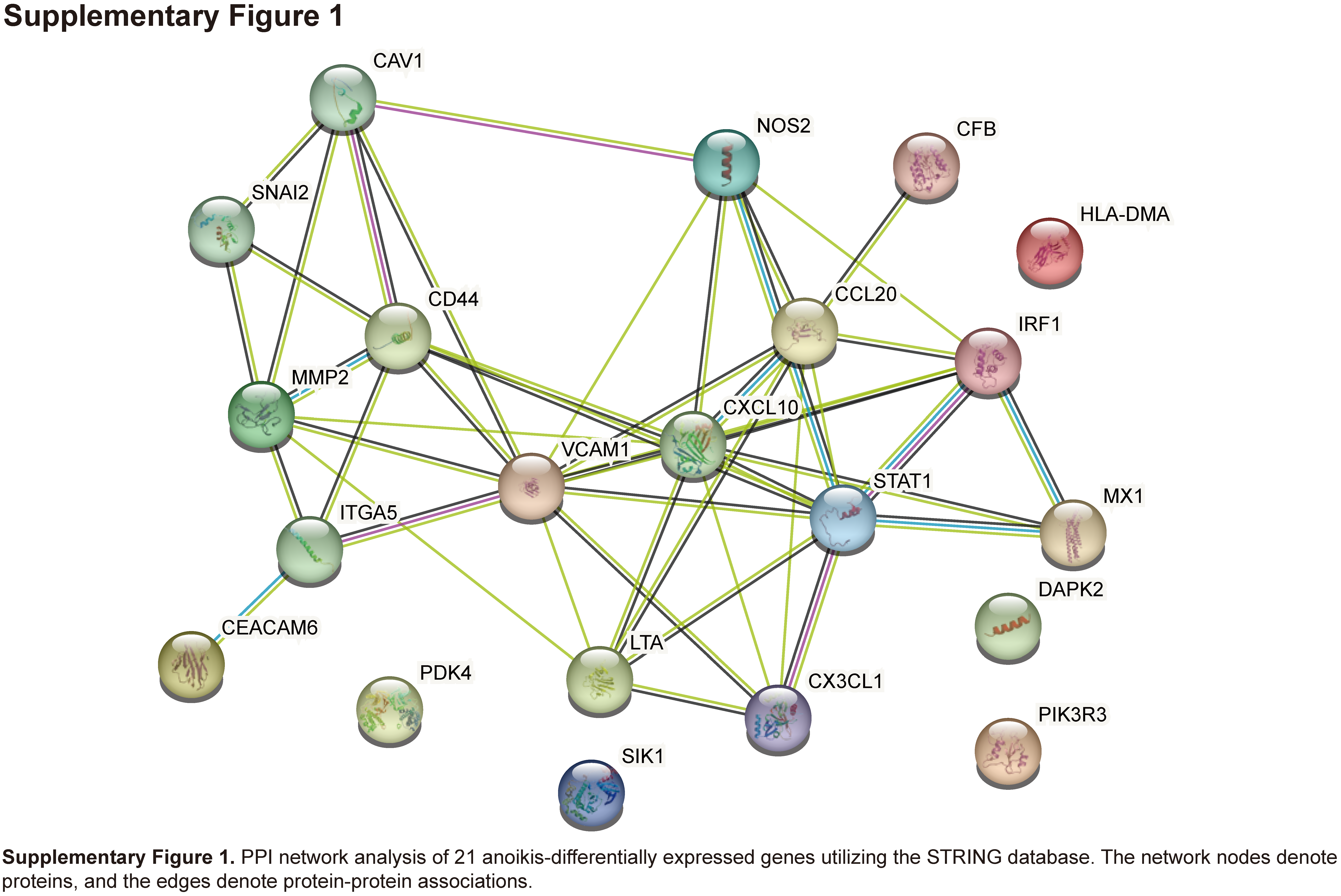

Supplement: Supplementary file 1 [file Image_1.TIFF]

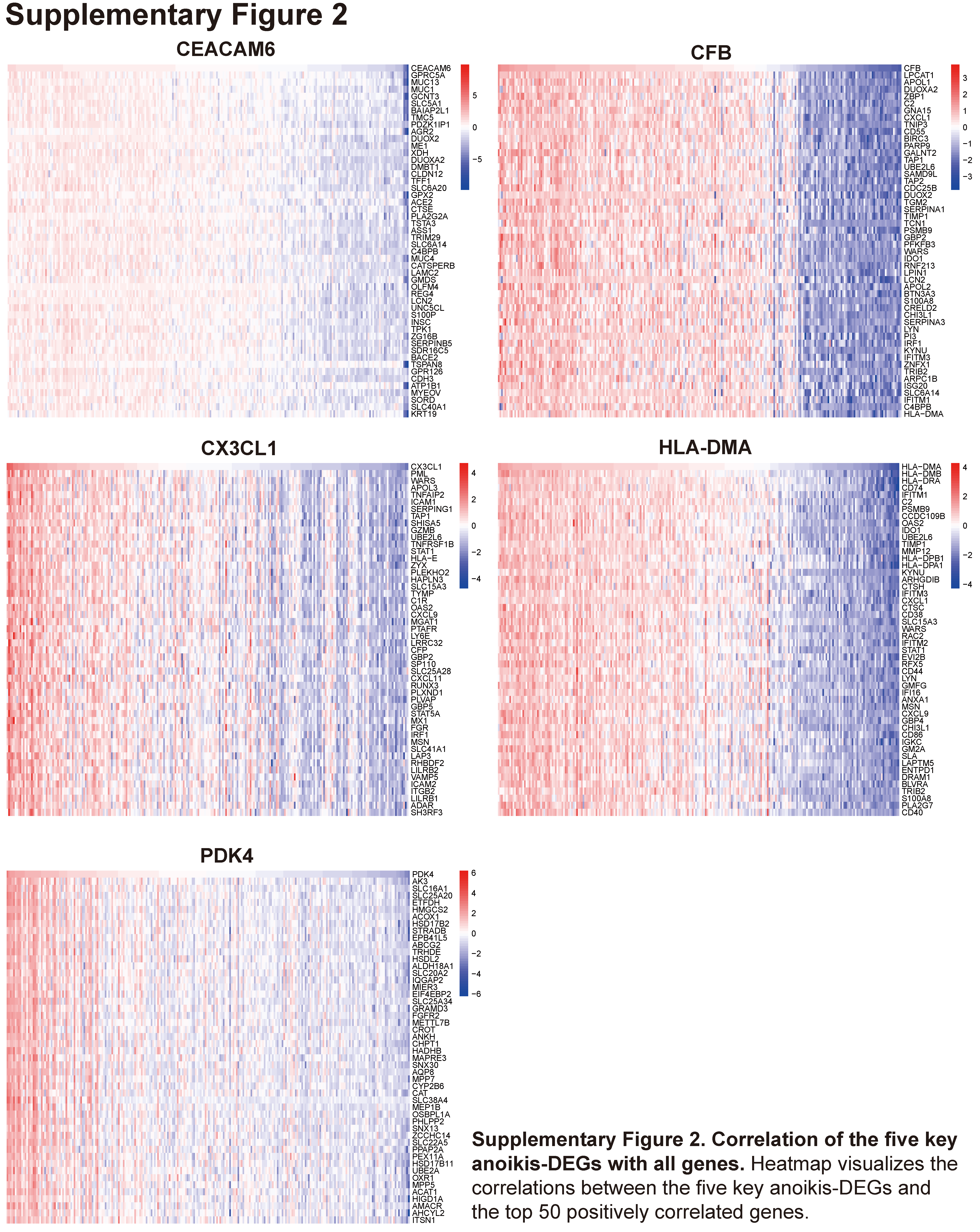

Supplement: Supplementary file 2 [file Image_2.TIFF]

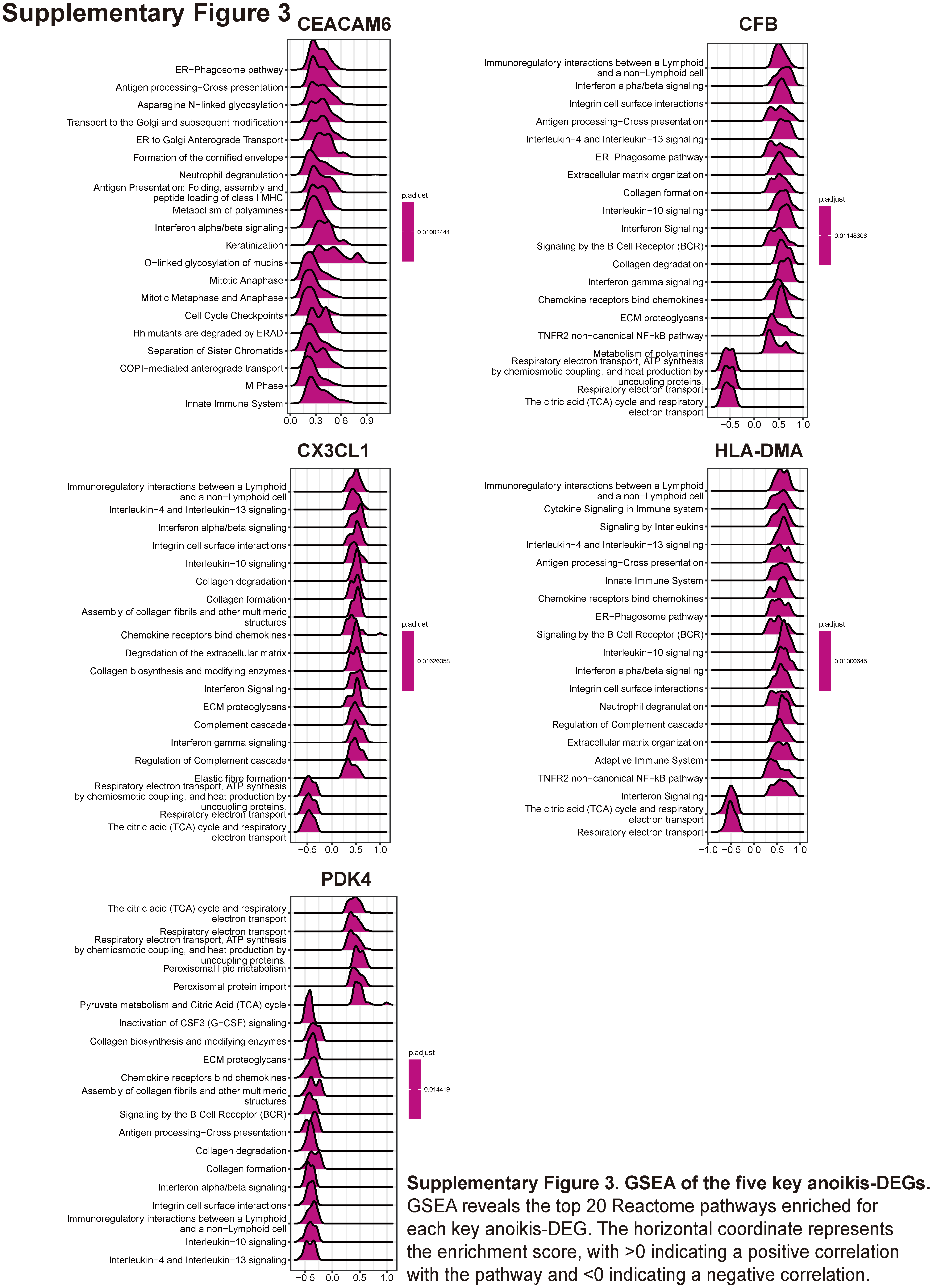

Supplement: Supplementary file 3 [file Image_3.TIFF]

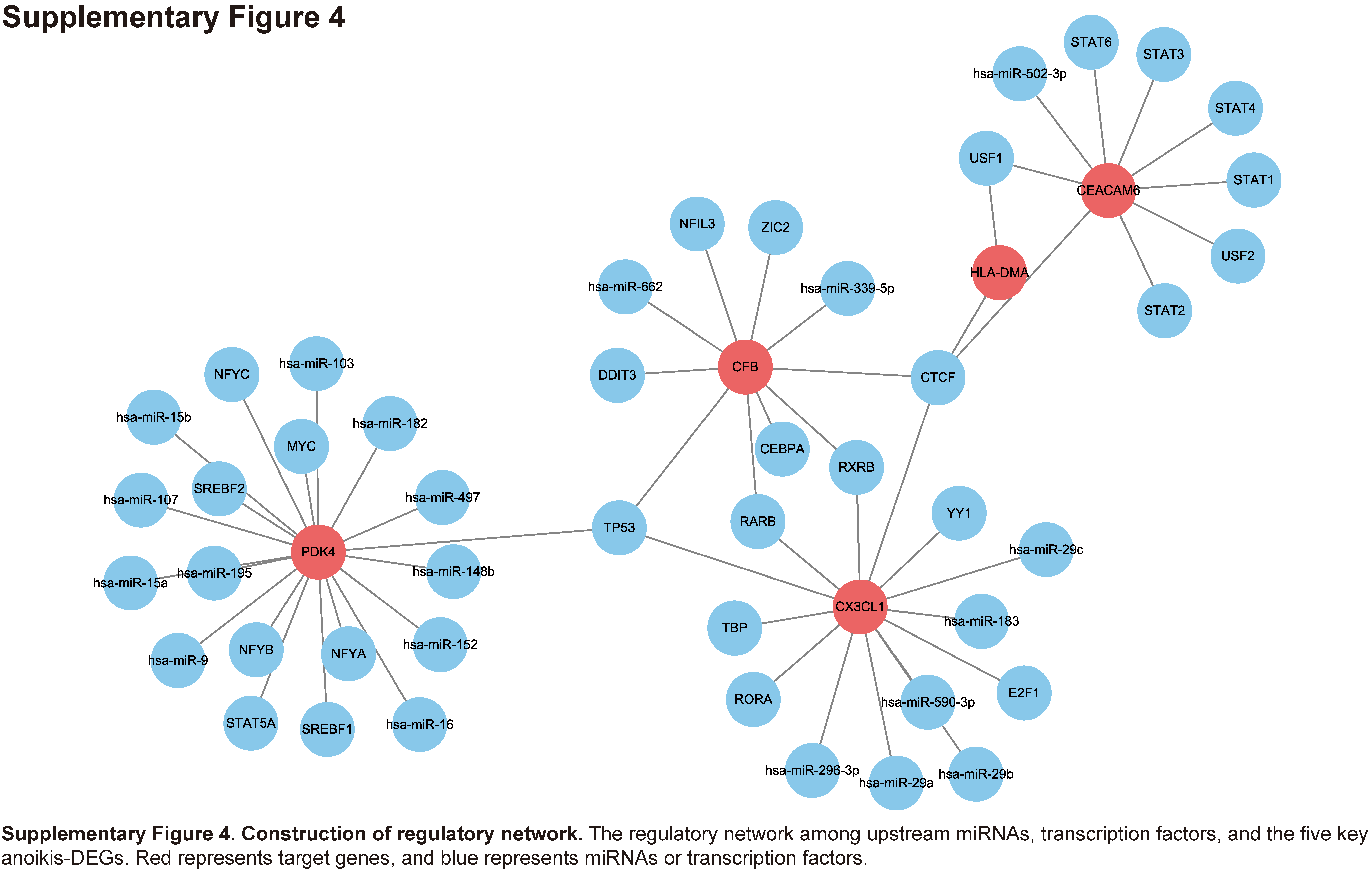

Supplement: Supplementary file 4 [file Image_4.TIFF]

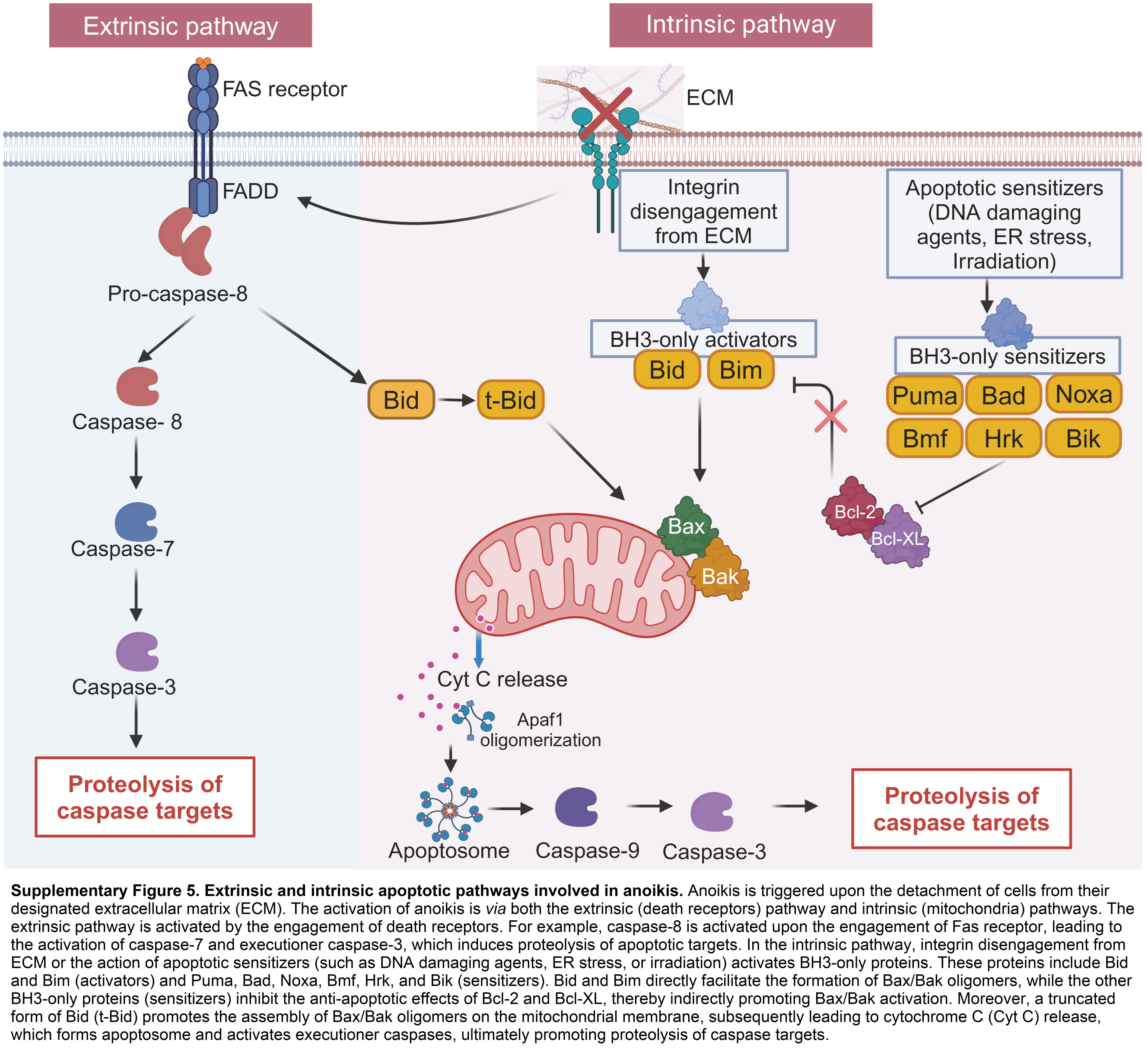

Supplement: Supplementary file 5 [file Image_5.TIFF]
